# Lymphoma survivors’ experience of participation in a home-based intervention post-chemotherapy

**DOI:** 10.1007/s11136-019-02244-3

**Published:** 2019-07-04

**Authors:** Suchita Hathiramani, R. Pettengell, H. Moir, A. Younis

**Affiliations:** 1 Faculty of Health, Social Care & Education, Kingston and St. George’s University of London, Cranmer Terrace, London, UK; 2 Haematology and Medical Oncology, St. George’s Hospital, St. George’s Healthcare NHS Trust, Tooting, London, UK; 3 Faculty of Science, Engineering and Computing, Kingston University London, Kingston upon Thames, Surrey UK

**Keywords:** Lymphoma survivors, Self-management, Experience, Service needs

## Abstract

**Purpose:**

Further research on patient experience and involvement is recommended in order to develop evidence-based and meaningful care pathways for lymphoma survivors. This study aims to explore the experience of a sample of lymphoma survivors participating in a home-based intervention following chemotherapy.

**Methods:**

Eligible participants who completed a 12-week home-based intervention were invited to complete the End of Study Questionnaire designed to explore perceptions, preferences and barriers to participation. Content analysis was used to generate codes, describe frequencies and identify themes.

**Results:**

Participating in a home-based intervention post-treatment was a positive experience overall, and aided recovery in this sample of lymphoma survivors (*n *= 35). Participants felt the programme provided structure, motivation and liked contact with the researcher. Participants highlighted their need for advice on healthy lifestyle, diet in particular.

**Conclusions:**

Lymphoma survivors in this study reported participation in a home-based intervention following treatment beneficial and aided recovery.

**Implications for cancer survivors:**

A large proportion of lymphoma survivors would benefit from a rehabilitation intervention post-chemotherapy. Intervention programmes should include follow-ups to monitor progress and provide support and motivation. Health professionals should recommend healthy lifestyle guidelines to survivors on completion of treatment or refer patients to appropriate services for rehabilitation and advice.

## Introduction

Like survivors of other cancers, the transition period from active treatment to survivorship can be challenging for lymphoma survivors, and they experience ongoing needs [1, 2]. Lymphoma survivors commonly report long-term and late effects of treatment including both physical and psychosocial symptoms such as fatigue, pain, muscle weakness, neuropathies, depression, anxiety, decreased function and quality of life (QoL) [3–5]. Interventions such as exercise and relaxation have been studied in cancer survivors and such programmes can have a positive effect on various symptoms including fatigue, strength, pain, stress, anxiety and quality of life [6–9].

Authors have highlighted the importance of further qualitative research in this field, as such data would capture the experience of rehabilitation as a whole, and aid understanding of potential factors which may influence preferences, motivators and barriers to participation [10–12].

This study aims to explore a sample of lymphoma survivors’ experience of participating in a home-based intervention post-chemotherapy. This study is part of the Relaxation and Exercise In Lymphoma (REIL) study; the aims and methods of the REIL study including background, design, eligibility, outcome measures and details of the home-based interventions are described elsewhere [13]. In this paper, we report the results from the End of Study (EOS) questionnaire following recommended Standards for Reporting Qualitative Research (SRQR) [14].

## Methods

### Study design

Data from the EOS questionnaire was collected between December 2014 and March 2017. Approval was obtained from Camden and Islington National Research Ethics Service (13/LO/1327), and St. George’s Hospital Joint Research and Enterprise Office (13.0108). The study is registered on a publicly accessible database, ClinicalTrials.gov (NCT02272751).

This study adopted a qualitative descriptive approach as described by Sandelowski [15, 16].

### Participants

Participants were recruited from the Haematology-Oncology Out-Patient Clinic at St George’s Hospital, London. Eligibility criteria included a diagnosis of lymphoma and in remission following primary treatment, completed chemotherapy within the last 6 weeks, age 18 years or older. Written informed consent was obtained from all participants, and participants informed that they could withdraw at any time.

### Procedure

EOS questionnaires were mailed to participants to complete at home, a stamped-addressed envelope included for return. The questionnaire comprised six open-ended questions—questions were not followed by any choice of replies, space was provided to give freedom to respondents and obtain their thoughts in their own words.

### Analysis

Content analysis [16] was used to analyse completed questionnaires. Transcribed questionnaires were read several times to derive common codes. As completed questionnaires were returned and codes applied, the researcher (SH) went back to the original transcripts to check reliability of previous codes and this process was repeated. When all completed questionnaires had been returned, similar codes were grouped into categories.

### Rigour

All steps in the analysis process were documented to ensure a clear audit trail. Reflections on reflexivity such as potential assumptions were discussed with the third author (HM). The researcher met with a researcher independent to the study (DT) to discuss preliminary codes and categories until consensus was reached and themes were agreed upon. Relevance and context of codes are demonstrated through quotes and extracts from data.

## Results

46 participants were invited to complete the EOS questionnaire, 35 participants (76%) completed and returned the questionnaire. The other 11 participants were lost to follow-up.

Respondent demographics are shown in Table 1. Results by theme are reported below.

**Table 1:** Demographic characteristics of respondents (*n *= 35)

	Number	Percentage
Gender		
Male	12	34.3
Female	23	65.7
Marital status		
Married	27	77.1
Single	3	8.6
Divorced	0	0
Widowed	5	14.3
Race/ethnicity		
White	31	88.6
Mixed race	0	0
Black/African/Afro-Caribbean	1	2.85
Asian	2	5.7
Other	1	2.85
Employment status		
Full-time	4	11.4
Part-time	2	5.7
Home-maker	4	11.4
Retired	18	51.4
Unemployed	1	3
Disability/sick leave	4	11.4
Other	2	5.7

### Positive experience

The vast majority (*n *= 30, 85.7%) found participation in a 12-week home-based intervention programme post-chemotherapy a positive experience, and reported they felt it helped recovery.

Codes emerged included ‘Encouraged’, ‘Gave focus to recovery’, and ‘Good to track progress’.

### Negative experience

A small number of respondents (*n *= 5, 14.3%) reported they did not feel it helped as they had few or no problems following treatment. Codes included difficulty finding time due to work and other commitments.

Positive and negative themes are summarised in Table 2.

**Table 2:** Positive and negative themes including codes, frequencies and quotes

Response	Code	Theme	Frequency	%
Relaxation intervention (*n *= 18)				
“It encouraged me and felt like a good back-up after the chemo.” (Female, 63)	Encouraged	Positive	15	83.3
“I think it was very helpful to have a ‘post-treatment assignment’. I think it helped create a bridge between chemo and ‘normal’ life, I’m really glad I participated.” (Female, 48)	Gave focus to recovery			
“I liked the regular contact with the person conducting the research as it made me feel like there were things being done to measure my recovery.” (Female, 39)	Good to track progress			
“It was helpful but I feel it went on too long, 8 weeks would have been enough.” (Female, 91)	Too long	Negative	3	16.7
“I found it increasingly difficult as I didn’t feel benefit from the relaxation and breathing exercise and they became a chore.” (Female, 39)	Difficult to do			
Exercise Intervention (*n *= 17)				
“It encouraged me to become active again after 6 months of ‘hibernation’.” (Male, 68)	Encouraged	Positive	15	88.2
“I enjoyed it, as it gave me a focus and ability to record what I had achieved.” (Male, 57)	Gave focus to recovery			
“It helped me find some support to carry on. I knew I was not alone somebody was looking after me also my body and see how I was progressing”. (Female, 61)	Good to track progress			
“My only regret was not being able to carry out all the exercises as suggested. As a carer for my wife I didn’t have the time to do it.” (Male, 51)	Took time	Negative	2	11.8
“Participation limited because of side effects in early stages of programme.” (Male, 72)	Difficult to do			

### Transition phase

Many respondents highlighted the difficulty of the transition phase:

I felt rather abandoned. (Female, 39).

After treatment there was a ‘hiccup’. I felt tired and found it annoying and frustrating. (Male, 57).

### Motivators

Participants particularly enjoyed the structure the programme provided:

I would have focused on activity anyway, but this programme helped—it gave me a structure and showed how much I needed to do. (Male, 57).

Having a designated activity with a set time encouraged me. (Female, 63).

Respondents also reported they liked the contact with the researcher and tests to measure fitness and recovery.

### Suggestions for improvement

The most common theme to emerge was the need for additional advice:

I don’t think the consultants were very clear or good at explaining how you could help them help yourself. Their advice was very vanilla, so impossible to do anything with. (Male, 57).

I would have liked advice on what you might expect—things that might happen that don’t mean anything is wrong just a result of chemo. Doctors are quick to say everything is ok, ‘be positive’. (Female, 77).

In particular, participants highlighted a need for dietary advice:

Advice on diet and dietary supplements would be helpful. There was no mention of this and I find that very disappointing. (Male, 60).

Would have liked some diet advice confirming what one is doing is on the right track. (Male, 53).

## Discussion

The aim of this study was to explore lymphoma survivors’ experience of participation in a home-based programme post-chemotherapy, and this was positive overall (85.7%). Studies have found lack of motivation to be the biggest psychological barrier to exercise participation in cancer patients [17–19]. Respondents in this study reported they felt that participating in the programme with regular visits to assess progress provided motivation to work towards recovery; and that they wouldn’t have done so otherwise. These findings support the need to address lack of motivation in lymphoma survivors by providing a structured programme, and support when required.

Luoma et al. [10] also explored participants’ experience of participating in an intervention post-treatment. Participants reported an increased sense of security with extra medical assessments and follow-up, similar to the current study. Requiring further support when transitioning off treatment, monitoring and measuring progress, and the importance of an expert instructor have also been highlighted as motivators to participation in post-treatment interventions in other qualitative studies [18, 20].

A small proportion of participants in this study reported that they did not find participation beneficial (14.3%) and did not adhere to the programme. This was due to one of two factors—either they could not find time to undertake the intervention, especially when returned to work; or they reported having minimal to no side effects from chemotherapy. A large proportion of survivors now return to work, and there is a need for further studies to look into developing interventions survivors are able to fit around returning to work [10, 21]. As some participants felt that they were back to ‘normal’ post-treatment, interventions may not be appropriate for every lymphoma survivor. A screening programme to determine which patients would benefit—either from a physical rehabilitation programme or psychosocial rehabilitation, or both—may maximise effectiveness of an intervention programme as routinely referring every patient may result in non-adherence by those who feel it is not required, as demonstrated here.

Another theme to emerge was the need for information, in particular advice on diet. A survey of 230 cancer survivors [22] also showed that almost all respondents (98%) desired further information following treatment, including diet and exercise. It has been pointed out that after completing medical treatment, survivors report that they are more likely to learn about cancer-specific information on their own, rather than from medical personnel [23]. Other qualitative studies of lymphoma survivors have also highlighted the lack of support and information during the transition phase [1, 2]. Results from this study further highlight the need for ongoing contact with a health professional and advice on healthy diet and lifestyle to be made available to lymphoma survivors following treatment.

Limitations of this study include recruitment from a single centre and potential selection bias. However, this study is one of the first to report lymphoma survivors’ own experience of undertaking a home-based intervention, and these preliminary findings offer insight into post-treatment experiences and support needs. Such findings will aid the development of meaningful and effective care pathways for lymphoma survivors.

## Conclusions

Participating in a home-based programme following treatment was a positive experience and aided recovery to pre-morbid function in this sample of lymphoma survivors. Participants felt the programme provided the support they required when care from the oncology team was suddenly decreased, and contact with the researcher provided encouragement, motivation and expert advice on how to progress recovery. A large proportion reported they did not receive sufficient advice on completion of treatment, and felt that advice on healthy lifestyle and dietary advice in particular was needed.

## Implications for cancer survivors

Results from this qualitative study indicate that a large proportion of lymphoma survivors would benefit from a home-based intervention programme following chemotherapy. Health professionals should recommend healthy lifestyle guidelines to lymphoma survivors, or refer patients to appropriate services for rehabilitation and advice.
